# Nutrients, Infectious and Inflammatory Diseases

**DOI:** 10.3390/nu9101085

**Published:** 2017-09-30

**Authors:** Helieh S. Oz

**Affiliations:** Department of Physiology, Internal Medicine, College of Medicine, University of Kentucky Medical Center, Lexington, KY 40536-0298, USA; hoz2@email.uky.edu

A balanced diet with sufficient essential nutritional elements is critical for maintaining a healthy body. Both nutritional excess and deficiency are associated with disease. For example, nutritional excess, particularly in refined carbohydrates and saturated fats, coupled with physical inactivity, can result in chronic inflammatory conditions such as obesity and cardiovascular disease. On the other hand, deficiencies in essential nutrients can lead to stunted growth, poor immune function and classical conditions such as scurvy, osteoporosis, depression and xeropththalmia. The gastrointestinal (GI) track takes in food and water, digests the food, extracts the nutrients and expels undigested/unabsorbed material as waste. Nutrients such as amino acids, oligosaccharides, and short-chain fatty acids have been recognized to be beneficial to the GI track and to human health in general, and they participate in shaping the immune system and in energy metabolism [1,2]. Short-chain fatty acids (acetate, propionate, and butyrate) are also produced naturally by the intestinal microbiome acting on prebiotics such as oligosaccharides and other indigestible fermentable fibers [3]. GI infection by microbial, viral or parasitic agents alters the gut microbiome and increases permeability to toxins. The microbiome is further altered by ingested antibiotics to treat bacterial infections. Microbial invasion stimulates inflammation, a defensive mechanism of the body’s immune system. This helps clear the invading microorganisms. However, persistent and excessive inflammatory response is a significant risk factor for developing various chronic inflammatory conditions and cancer, and increases the risk of succumbing to infectious diseases, owing to T cell exhaustion [4] The collection of articles in this issue have been compiled to help illuminate the contribution nutrients make to the prevention, treatment and taming of a range of inflammatory and infectious diseases.

Chronic inflammatory diseases affect millions of people globally. While inflammation contributes to the tissue healing process, chronic inflammation can lead to the loss of tissue function and organ failure. Chronic inflammation which accompanies conditions such as chronic hepatitis, inflammatory bowel disease and neurodegenerative disorders increases the risk of malignancy. Despite rapid advancement in diagnostics and the availability of therapeutic options, there remains no effective cure for patients who suffer from inflammatory diseases. Therefore, patients seek alternatives and complimentary agents as adjunct therapies to relieve symptoms and possibly prevent consequences of inflammation. Oz H.S. [5], has investigated the anti-inflammatory properties of green tea polyphenols (GrTPs) with potent antioxidant activities in a variety of settings. These include their ability to inhibit the I-κB kinase nuclear factor-kappa B (NF-κB) signaling pathway, induction of programmed cell death (caspases, Bcl-2), release of inflammatory cytokines and production of lipid mediators of the cyclooxygenase (Cox) system. The author reviews relevant investigations regarding protective and adverse effects as well as possible applications for GrTPs in the treatment of chronic and inflammatory complications. GrTPs also possess antimicrobial properties, including the ability to inhibit the growth of *Mycobacterium Tuberculosis* (TB) in macrophages [6] Worldwide, approximately 2–3 billion people are infected with TB and 5–15% of these individuals will develop some form of active TB. Using a structured questionnaire, Soh A.Z. et al. [7] investigated the effect of drinking black or green tea, or coffee on the risk of TB activation in a prospective population-based cohort involving 63,257 tea-drinking Singaporean Chinese. With a mean follow-up period of 16.8 years, the authors reported that drinking black or green tea was associated with a dose-dependent reduction in the risk for TB infection. This association was not evident with coffee or caffeine intake. The authors concluded that regular tea drinking is associated with reduced risk of active TB.

Inflammatory bowel disease (IBD), predominantly Crohn’s disease and ulcerative colitis, affects 1.8 million people in the US and there is no available cure. Although environmental factors have been implicated in the etiology of IBD, a breakdown in immune homeostasis, caused by a dysregulated adaptive immune response against intestinal bacterial flora in genetically predisposed individuals, is believed to be a key pathogenetic factor [8,9]. Crohn’s disease is frequently a progressive disease. Up to half of these patients require surgical interventions within about 10 years of diagnosis and over 75% of these operated patients require at least one further surgery in their lifetime. Currently, guidelines for nutrition in general surgery also apply to Crohn’s patients. To minimize the risk of surgery, it is necessary to optimize the nutritional status for these patients. The systematic review by Grass F. et al. [10] investigated preoperative nutritional support in adult Crohn’s patients between 1997 and 2017 and aimed to overview screening modalities, routes of administration and expected benefits in these patients. They selected 29 studies of which were original and 15 were review articles. Malnutrition was found to be a major risk factor for postoperative complications, and both enteral and parenteral routes were efficient in decreasing postoperative morbidity. The authors recommended that the route of administration should be chosen based on disease presentation and the patients’ condition. Further studies are needed to strengthen these evidences. Further, IBD patients may be at risk of experiencing vitamin B (Vit B) and folate deficiencies owing to malabsorption in the IBD-affected intestines. However, an association between IBD and serum folate and Vit B12 concentrations remains controversial. A multiple database meta-analysis performed by Pan Y. et al. [11] to compare serum folate and Vit B12 concentrations in IBD compared to controls revealed that the average serum folate concentration in IBD patients was significantly lower than controls. Interestingly, this difference was only observed with ulcerative colitis patients, but not with Crohn’s disease patients. No difference was detected for the mean serum Vit B12 levels. The authors concluded that folate deficiency might play a role in the development of IBD, although the data did not indicate causation. The authors suggested that folate and Vit B12 supplementation in IBD patients might improve their nutritional status and prevent other conditions. 

Dietary considerations are important in IBD as nutritional factors may be directly involved in the pathogenesis and recurrence of IBD. Furthermore, dietary factors may also influence the treatment of IBD. Exclusive enteral nutrition (EEN) has been shown to have beneficial effects in various conditions including in Crohn’s disease, and this has been recommended in Europe to be the first-line therapy for inducing remission in pediatric luminal Crohn’s disease [12,13,14]. However, the mechanism of action is elusive. One possibility is that EEN could alter the microbiota in the patients. Gatti S. et al. [15] reviewed 14 different clinical trials, involving 216 Crohn’s patients, which investigated the effect EEN had on the microbiota. Interestingly, patients on EEN had a profound decrease in microbiota diversity, which switched back to their natural status upon conclusion of EEN. The EEN appeared to cause metabolomic changes. Despite the interesting finding, inconsistencies were detected between studies in the effect EEN had on specific bacterial strains and this awaits further microbiological analysis using newer techniques such as next-generation DNA sequencing.

Probiotics and synbiotics are used to treat chronic inflammatory diseases such as IBD. The probiotics and synbiotics effects have been studied on chronic intestinal diseases in in vitro animal models and in humans in randomized clinical trials. Probiotic strains and their cell-free supernatants reduce the expression of pro-inflammatory cytokines via actions which are principally mediated by toll-like receptors. Probiotic administration improves clinical symptoms, histological alterations, and mucus production in most of the evaluated animal studies. Probiotic supplementation appears to be well tolerated, effective and safe in IBD patients. For instance, *Bifidobacterium longum* improved clinical symptoms in patients with mild to moderate active colitis. However, some results suggest that caution should be taken when administering these agents in the relapsed stages of IBD. In addition, no effects are reported on chronic enteropathies. Consequently, although probiotics are shown to provide benefits, Plaza-Díaz J [16] suggested that the risks and benefits should be carefully assessed before initiating therapy in these patients. Further studies are required to understand the exact mechanism by which probiotics and synbiotics affect these diseases.

Obesity has become a global problem. One of the emerging issues is the link between high pre-pregnancy body mass index (BMI) and an increased risk of adverse pregnancy outcomes. There have been limited studies regarding the relationship between pre-pregnancy BMI and the inflammatory potential of the diet during pregnancy. Shin D. et al. [17] included 630 pregnant women from the U.S. National Health and Nutrition Examination Survey (NHANES) with cross sectional examinations from 2003 to 2012. Pre-pregnancy BMI was calculated based on self-reported weight and measured height. The authors reported that women with pre-pregnancy obesity (high-BMI) are more likely than those with normal weight to have high dietary Inflammatory Index and elevated levels of C-reactive protein (CRP).

Micronutrient homeostasis is a key factor in maintaining a healthy immune system. Zinc (Zn) is an essential micronutrient that is involved in the regulation of the innate and adaptive immune responses. Malnutrition is the main cause of Zn deficiency which leads to cell-mediated immune dysfunctions and other manifestations [18]. Consequently, immune dysfunction leads to a worse outcome toward bacterial infection and sepsis. Zn is required for pathogen-eliminating signal pathways leading to neutrophil extracellular traps formation, as well as cell-mediated immunity over humoral immunity. Zn deficiency plays a role in inflammation to damage the host tissues. Zn is involved in the modulation of the proinflammatory response by targeting NF-κB, a transcription factor that is the master regulator of proinflammatory responses. It is also involved in controlling oxidative stress and regulating inflammatory cytokines. Zn is critical for sustaining proper immune function. Gammoh N.Z. and Rink L. [19] review the role of zinc and its deficiency during infections and inflammatory responses and modulation of the immune system.

Copper (Cu) is another essential trace element that is required for development. Infections alter Cu and Zn metabolism, deficiencies of which can increase infection risks. Wisniewska M. et al. [20] conducted a prospective observational case-control study in 21 infected and 23 control term and preterm newborns. Median concentration of Cu at birth (day 1) was 522.8 μg/L, and Zn was 1642.4 μg/L. Cu and Zn correlated positively with gestational age in control newborns. The authors concluded that infections affect the trace element homeostasis in newborns; while serum concentration of Zn is reduced, Cu and CRP levels are increased. The Cu/Zn ratio may constitute of a meaningful diagnostic biomarker for early-onset of infections. Selenium is a specific trace element and essential for normal metabolism. Minor selenium deficiency is accompanied by health defects whereas severe deficiencies are associated with immunodeficiency, affecting both cell-mediated and humoral immune functions. Selenium supplementation improves immune function in depleted individuals. Pregnant women and infants are at risk for selenium deficiency, with negative effects on immune and brain function. Varsi K. et al. [21] investigated selenium levels in two different groups: (1) 158 healthy women who had never had any children; and (2) 140 women with singleton pregnancy who were followed from pregnancy week 18 through to 6 months postpartum. Prevalence of infant infection was reported by the mothers from 6 weeks to 6 months of age and neurodevelopment of the infants were assessed using the Parental Questionnaire Ages and Stages at 6 months of age. The authors reported that low maternal selenium status (≤0.78 μmol/L) at week 36 of pregnancy was associated with an increased risk of infection in the babies during the first 6 weeks of age and low maternal selenium status (≤0.90 μmol/L) in pregnancy week 18 was associated with a lower psychomotor growth scores at 6 months of age. The authors recommended that maternal serum selenium should be greater than 0.90 μmol/L in pregnancy week 18 and 0.78 μmol/L in pregnancy week 36 to benefit the infants.

Chronic pancreatitis leads to pancreatic cancer, one of the most aggressive forms of cancer. Since pancreatic cancer cell lines express high levels of insulin-like growth factor (IGF-I and the IGF-I receptors that stimulate cell proliferation, angiogenesis and the invasiveness of cancer cells [22,23] it is possible that IGF-I and related growth factors could play a role in promoting pancreatic cancer. Gong Y. et al. [24], in a meta-analysis study, investigated the association between serum concentration of IGF-I and the risk of pancreatic cancer. Ten studies, published between 1997–2013, which met their inclusion criteria were selected from Medline and EMBASE databases. The authors found no correlation between serum concentrations of IGF-I and -II, IGFBP-1 and -3, and IGF-I/IGFBP-3 ratio with risk of pancreatic cancer. Thus, serum IGF-I, IGF-II, IGFBP-1 and IGFBP-3 as well as the IGF-I/IGFBP-3 ratio may not be associated with the development of pancreatic cancer. Further studies are required to confirm these findings. 

Ginseng is an herbal supplement with a wide-range of medicinal effects and low side effects. Ginsenoside (Rg1) is one of the major active ingredients of ginseng with some beneficial effects in neurodegenerative diseases such as Alzheimer’s disease. Furthermore, Rg1 has anti-inflammatory effects. Given these actions, it is possible that Rg1 may have therapeutic value against osteoarthritis, a condition that is characterized by degenerative changes and inflammatory responses in chondrocytes. Cheng W. et al. [25] assessed the anti-inflammatory effects of Rg1 in human chondrocytes and whether Rg1 was able to reduce articular cartilage damage in a rat model of osteoarthritis. The authors reported that Rg1 suppressed IL-1β-induced inflammatory responses in human chondrocytes and reduced disease activity in the joints of the rats. 

The microbiota affects the functioning of physiological, metabolic and immunological process. For instance, microbiota regulates the growth and functions immune cells in the intestines. Evidence indicates that alteration in the gut microbiota can influence infectious and inflammatory diseases. Bacteria that reside on the mucosal surface or within the mucosal layer interact with the host immune system. Thus, a healthy gut microbiota is essential for the development of mucosal immunity. In HIV patients and those with controlled viremia by using antiretroviral drugs their gut microbiome is different than those uninfected HIV controls. Recent data suggest the patients with dysbiosis may have a breakdown in their gut immunological activity, causing systemic bacterial diffusion and inflammation. Treating GI tract disorders become difficult tasks in HIV-infected patients and those on antiretroviral drugs. Therefore, trails are investigating the ability of probiotics to modulate epithelial barrier functions, microbiota composition, and microbial translocation. D’Angelo C. et al. [26] conducted a review on the use of probiotics to treat HIV-induced gastrointestinal tract disorders and in improving gut-associated lymphoid tissue (GALT) immunity. 

The beneficial properties of pistachio nuts have been extensively reported from pistachio polyphenolic extracts in nuts, resins and leaves. These effects include antioxidant and anti-inflammatory, antipyretic, antibacterial and antiviral, and are used for to treat infections, eczema, asthma, kidney stones, diarrhea/GI complications and abdominal pain. Paterniti I. et al. [27] investigated the anti-inflammatory and anti-oxidative stress properties of polyphenolic extracts from “raw pistachios” and “roasted, salted pistachio” utilizing lipopolysaccharide (LPS) stimulated monocyte/macrophage cell line J774 and carrageenan induced inflammatory paw edema in rat model. The authors found anti-inflammatory and antioxidant properties of pistachio at lower doses than those reported before. The data support beneficial effects associated with consumption of pistachios.

Pomegranate (*Punicagranatum*), a phytochemical rich fruit, has been used for centuries to prevent and treat inflammatory conditions. Mandal A. et al. [28] previously reported that pomegranate extract inhibited dimethylbenz (a) anthracene (DMBA)-initiated rat mammary tumorigenesis by anti-proliferative actions and apoptosis. This is a continuation of their previous study to investigate the mechanism of anti-inflammatory action for pomegranate extract in the same model. The authors demonstrated that pomegranate emulsion was able to prevent DMBA-evoked mammary carcinogenesis though anti-inflammatory mechanisms by inhibiting NF-κB while upregulating Nrf2 signalling.

l-Arginine is a non-essential amino acid and nitrogen carrier for the synthesis of urea, polyamines, proline, and other proteins. Arginine has immune-regulatory and anti-inflammatory properties. Consequently, it is frequently administered to critically ill patients with sepsis. Yeh C-L and her group [29] investigated the influence of intravenous arginine administration to alter circulating proangiogenic cells and remote lung injury in mouse model of polymicrobial sepsis induced by cecal ligation and puncture. The authors demonstrated that arginine administration promoted mobilization of circulating proangiogenic cells while down-regulating the sepsis-induced production of inflammatory cytokines and expression of angiopoietin 1 and 2, and their receptor, Tie-2 mRNA in lungs. The authors suggested that more studies are required to determine whether their observations are involved in mediating the beneficial effects of arginine. 

Vitamin D (Vit D), a fat-soluble steroid and a pro-hormone, is produced endogenously in the skin by a direct action of ultraviolet sunlight, and a portion delivered through dietary intake. Two forms of Vit D supplements available over-the-counter are ergocalciferol (Vit D2) and cholecalciferol (Vit D3). Vit D2 is commonly added to foods; whereas, Vit D3 is mainly synthesized in the skin and present in food animal products. Numerous studies have suggested the importance of Vit D to protect against diseases including obesity and malignancies. Vit D production and its receptor have been reported in multiple tissues, with a vital role in promoting the immune system. Rickets, a defect in bone growth in children, due to Vit D deficiency, was first recognized in 1650. From 1930s, Vit D2 has been added to milk in U.S. and Europe to eliminate rickets. Vit D deficiency is highly prevalent throughout the world, affecting host immunity leading to an increased incidence and severity of several infectious diseases. Vit D deficiency may be caused by different drugs’ interaction including steroids, chemotherapies and lack of sunlight exposure. Yet, high doses of Vit D supplements are linked with an increased risk of fractures, kidney stones and certain cancers. Gois P.H.F. et al. [30] reviewed recent literature on the relationship between Vit D and the immune system, Vit D status and the risk of contracting infectious diseases such as TB, respiratory tract infections and fungal infections, and Vit D status and sepsis, and disease progression and mortality in human immunodeficiency virus-infected patients. In addition, the authors reviewed the outcomes of Vit D supplementation as a treatment/prophylaxis in the above mentioned diseases/conditions. Overall, there appears to be lack of agreement between results from these investigations. 

Dendritic Cells (DC) are vital for antigen presentation and initiation of an adaptive immune response against hostile infection as well as immune tolerance for harmless/beneficial microbiota. Food products can modulate the inflammatory status of intestinal DCs. Quercetin is a phytochemical (flavonoid), which can suppress the secretion of inflammatory cytokines, antigen presentation and DCs migration towards the draining lymph nodes. De Santis S. et al. [31] recently identified Secretory Leukocyte Peptidase Inhibitor (Slpi) expression to be required for quercetin to inhibit the secretion of proinflammatory cytokines and chemokines. Quercetin-enriched food was found to be able to induce Slpi expression in the ileum, while little effects are detectable in the duodenum. Slpi expressing cells are located at the tip of the intestinal villi, as quercetin exposure could be more efficient for DCs projecting “periscopes” in the intestinal lumen. The data suggest that quercetin could suppress inflammation in ileo-colonic tract.

Toxoplasmosis is a common foodborne and congenital disease, caused by *Toxoplasma gondii*, an apicomplexan microorganism which infects muscle, neural tissue, and brain, in humans and animals [32]. Toxoplasmosis has been reported to be associated with behavioral and cognitive modulation yet the mechanism of action is not well known. There is some evidence which suggests that *Toxoplasma* extracts folate from neurons. Since reduced folate availability is known to be associated with an increased risk of neurodevelopmental disorders, neurodegenerative diseases, and cognitive decline, Berrett A.N. et al. [33] analyzed data from the third National Health and Nutrition Examination Survey to determine the associations between *Toxoplasma* infection, multiple folate-cycle factors, and cognitive function in adults aged 20 to 59 years in United States. The data suggest that *Toxoplasma* infection affected levels of folate and/or Vit B-12 in brain to alter cognitive functioning.

Millions of people are infected with Hepatitis C virus (HCV) which may lead to hepatocellular carcinoma. The mechanism by which HCV infection affects adipokines in the host remains unclear. Chang M-L et al. [34] presented a prospective trial with 450 patients with genotype 1 and 2 who had completed anti-HCV therapy. Patients surveys were used to assess pre-therapy and 24-week post-therapy and levels of adipokines including leptin, adiponectin and plasminogen activator inhibitor-1 (PAI-1). The authors reported specific associations between some of these parameters and suggested that HCV infection could mask other associations e.g., between increased adipokines levels and metabolic and hepatic profiles. 

A major cause of admission into pediatric intensive care unit (ICU) is severe inflammatory/infections and sepsis. The metabolic response to stress and infection correlates with the severity of insult and energy requirement obtained from protein, fats and carbohydrates. The nutritional status in the admitted children tends to deteriorate during the course of the illness and this has a negative impact on clinical outcome. Thus, it is important to accurately determine the energy requirements in the ICU in order to avoid under- or over-feeding. De Cosmi V. et al. [35] discussed the metabolic changes in critically ill children and the notion for personalized pediatric nutritional interventions. The authors report the major role for macronutrients, blood glucose levels and the acute phase proteins by means of indirect calorimetric procedure. Authors concluded that personalized nutritional interventions in these patients are needed to use glucose/fat balance to reduce catabolic consequences in critical stages and speed their recovery. 

Parkinson’s disease is a neurological disorder characterized by neuroinflammation, loss of dopaminergic neurons within the midbrain. Due to insufficient therapies and adverse effects of conventional drugs, there is an urge for use of new unconventional interventions for the treatment of Parkinson’s. Atractylenolide-I (ATR-I) is a major bioactive ingredient isolated from the plant rhizomes of *Atractylodes Macrocephala*, also known as “Baizhu”, a traditional Chinese medicine used for anti-gastrointestinal dysfunctions and has anti-oxidant and anti-cancer activity. More S and Choi D-K [36] investigated the anti-neuroinflammatory mechanisms of ATR-I in in vitro and in vivo models of Parkinson’s disease. Intraperitoneal administration of ATR-I decreased microglial activation and protected dopaminergic neurons. In vitro, ATR-I inhibited NF-κB activation and enhanced the expression of hemoxygenase-1. The authors argue that ATR-I may be useful as a new therapeutic agent for Parkinson disease.

Neuromuscular diseases (NMDs) are a heterogeneous group of acquired or inherited syndromes. NMDs frequently accompany nutritional complications. Salera S. et al. [37] argued that with the prolongation of survival in patients with NMDs, it is important to consider nutritional issues in these patients. These include over-nutrition, glucose metabolism, mobility, respiratory and cardiologic functions. Hypo-nutrition affects muscles and ventilatory function, constipation and other GI disorders, chewing/swallowing difficulties as risk factors for aspiration which predisposes to infections, respiratory complications, osteoporosis and increased risk of fractures. Focusing on the care of children with Duchenne muscular dystrophy, the authors reported that appropriate nutritional care can improve the quality of life in these patients. Further studies are needed to investigate the relevance of over-nutrition and under-nutrition, GI, infections dysphagia and reduced bone mass in the different types of neuromuscular diseases and information on appropriate percentiles of weight, height, body mass index and body composition are critical for improved the management of these patients.

Respiratory tract infections are the most common infections in children and adults. Recurrent respiratory tract infections are prevalent mostly in early childhood, causing high indirect and direct costs on the healthcare system. Recurrent respiratory tract infections are usually the consequences of immature immunity in children and immunosuppressed complications in adults with high exposure to various respiratory pathogens. Biologically active polysaccharides like β-glucans are extensively studied as natural immunomodulators, anti-inflammatory, and anti-infectious activities. Jesenak M. et al. [38] reviewed the use of β-glucans as possible therapeutic and preventive approach in managing and preventing recurrent respiratory tract infections in children (β-glucans from *Pleurotus Ostreatus*), adults (yeast-derived β-glucans), and in elite athletes (β-glucans from *Pleurotus Ostreatus* or yeast).

Finally, a balance and vigorous gut microbiota is necessary to support health and growth in host. Overgrowth of gut microbial or pathogens can change ecosystem, and compromise gut integrity to initiate GI complications. So far there is no safe and effective modality against GI pathogenic coccidiosis. Antibiotic additives routinely fed to food animals to protect against infection, will inevitable enter the food chain, contaminate food products and pass to the consumers. A century after the original discovery of poultry coccidiosis, Oz H.S. [39], introduces mechano-chemically altered coccidial organisms with distinct ultrastructures, but without abolishing their immunogenicity. These aberrant organisms were tolerated by cyclophosphamide immunodeficient-animals yet were non-pathogenic, and provide novel immune-protection in immune-intact-animals against pathogenic challenges which included diarrhea, malnutrition and weight loss. This study warrants further investigations toward vaccine production. In conclusion, this Special Issue includes a collection of innovative articles from basics, translational findings and clinical trials as well as reviews into the relationship between infectious/inflammatory diseases and nutrient. Prospective clinical trials, novel diagnostic, preventive and therapeutic modalities which are discussed can aid the development of nutritional strategies for the treatment as well as prevention of inflammation and infection. Finally, the original reviews are of particular interest to help in advancing our understanding in signaling pathways, molecular and biochemical mechanisms behind the effects of nutrients on inflammatory and infectious diseases. Different nutritional and dietary life styles, whether poor or lacking essential nutritional elements, as well as excess intake, can result in inflammatory complications and loss of function. Nutritional deficiency is linked with several infectious and inflammatory diseases as a cause or consequence. Studies indicate nutrients, such as amino acids, oligosaccharides, and short-chain fatty acids exert inhibitory and anti-inflammatory functions. Gastrointestinal (GI) infections alter gut microbiome and increase permeability to toxins. Various invasions by microbial, viral and parasitic agents stimulate inflammation, a defensive mechanism of the body’s immune system. Other stimuli include environmental, oxidative stress, aging and the physiological process. Long-lasting persistent and excessive inflammatory response is a significant risk factor for developing various chronic inflammatory and infectious diseases. The following investigations may help to understand nutritional contributions in the prevention, treatment and to tame certain inflammatory and infectious diseases.

## Abbreviations

ATR-I	Atractylenolide-I
BMI	body mass index
Vit D3	cholecalciferol
DC	Dendritic Cells’
Vit D2	Ergocalciferol
EEN	Exclusive Enteral Nutrition
GI	gastrointestinal
HCV	Hepatitis C virus
IBD	Inflammatory bowel disease
IGF	insulin-like growth factor
ICU	Intensive care unit
LPS	lipopolysaccharide
NMDs	Neuromuscular diseases
PAI-1	plasminogen activator inhibitor-1
vit	Vitamin
RTIs	Respiratory tract infections
RV	Rotaviruses
*Toxoplasma*	*Toxoplasma gondii*
